# Honest Boys Make Honest Men

**Published:** 1879-10

**Authors:** 


Honest boys make honest men, and honest men make happy homes, good citizens, fair dealers, true Christians, and just legislators; while dishonesty fills the land with sufferings and wickedness, and peoples jails and prisons with both young and old. Could granite walls and iron bars speaks to us, they would tell us the sad fate of thousands who began their downward career by deceiving and lying. This is a wicked and perverse generation, and honest men are hard to find. Children, if you wish to shun the downward road to ruin; if you wish to be successful and respected; if you wish to make glad the hearts of your parents; and above all, you wish to please your heavenly Father, tell the truth, for "Lying lips are abomination to the Lord; but they that deal in truth are His delight."—*Watchman and Reflector.*

